# Nondestructive and rapid determination of lignocellulose components of biofuel pellet using online hyperspectral imaging system

**DOI:** 10.1186/s13068-018-1090-3

**Published:** 2018-04-02

**Authors:** Xuping Feng, Chenliang Yu, Xiaodan Liu, Yunfeng Chen, Hong Zhen, Kuichuan Sheng, Yong He

**Affiliations:** 1 College of Biosystems Engineering and Food Science, Zhejiang University, Hangzhou, 310058 China; 2 Vegetable Research Institute, Zhejiang Academy of Agricultural Sciences, Hangzhou, 310021 China

**Keywords:** Hyperspectral imaging, Image processing analysis, Biofuel pellet, Lignocellulose components, Wavelength selection, Biomass

## Abstract

**Background:**

In the pursuit of sources of energy, biofuel pellet is emerging as a promising resource because of its easy storage and transport, and lower pollution to the environment. The composition of biomass has important implication for energy conversion processing strategies. Current standard chemical methods for biomass composition are laborious, time-consuming, and unsuitable for high-throughput analysis. Therefore, a reliable and efficient method is needed for determining lignocellulose composition in biomass and so to accelerate biomass utilization. Here, near-infrared hyperspectral imaging (900–1700 nm) together with chemometrics was used to determine the lignocellulose components in different types of biofuel pellets. Partial least-squares regression and principal component multiple linear regression models based on whole wavelengths and optimal wavelengths were employed and compared for predicting lignocellulose composition.

**Results:**

Out of 216 wavelengths, 20, 10 and 17 were selected by the successive projections algorithm for cellulose, hemicellulose and lignin, respectively. Three simple and satisfactory prediction models were constructed, with coefficients of determination of 0.92, 0.84 and 0.71 for cellulose, hemicellulose and lignin, respectively. The relative parameter distributions were quantitatively visualized through prediction maps by transferring the optimal models to all pixels on the hyperspectral image.

**Conclusions:**

Hence, the overall results indicated that hyperspectral imaging combined with chemometrics offers a non-destructive and low-cost method for determining biomass lignocellulose components, which would help in developing a simple multispectral imaging instrument for biofuel pellets online measurement and improving the production management.

**Electronic supplementary material:**

The online version of this article (10.1186/s13068-018-1090-3) contains supplementary material, which is available to authorized users.

## Background

Exploration and utilization of biomass energy has received much attention because of the problems caused by energy shortages and environmental pollution. There is a growing market for producing compressed biofuel pellets from forest and agriculture residues since this biofuel has advantages of easy storage, convenient transport and being environment friendly [1]. However, low energy-conversion efficiency and poor moisture resistance make the direct use of biofuel pellets less attractive. Hydrothermal carbonization (HTC) is a novel technology for rapid conversion of lignocellulosic biomass into carbon-rich and value-added products called hydrochar [2]. The different weight percentages of lignocellulose components and their physical architecture affect the fuel characteristics of hydrochar. For example, lignin is more difficult to degrade and decompose than hemicellulose or cellulose [3], and lignin is the main contribution to hydrochar solid yield [4]. Lignocellulosic composition and proportion varies by species and these and production conditions can significantly influence the process scheme. Therefore, it is necessary to accurately determine the lignocellulose components for further hydrothermal treatment. Unfortunately, the conventional chemistry approach for measuring the concentration of lignocellulose components in biomass is accurate, but time-consuming, cumbersome and expensive. These analyses require complex pre-treatment and some toxic chemical reagents, and are not suitable for the large-scale sample measurement needed for industry online application.

Near-infrared (NIR) spectroscopy is a reliable alternative to traditional methods for efficiently evaluating chemical components in a non-destructive manner [5]. The NIR spectral region (800–2500 nm) is dominated by the bands related to the overtones and combination of fundamental vibrations (e.g., N–H, C–H, O–H and S–H), which are the foundation for analysis of lignocellulose components in biomass [6]. Hodge et al. [7] developed global NIR models for rapid determination of cell wall components in pine wood which gave high correlation coefficients with independent validation of 0.97 for lignin and 0.82 for cellulose. Jin et al. [8] reported that hemicellulose, cellulose and lignin contents could be predicted with NIR spectroscopy and chemometric method for *Miscanthus sinensis* with excellent results. Xue et al. [9] applied NIR spectroscopy and a partial least-squares regression as a rapid and non-destructive method for online measurement of lignocellulose components of corn stover. In addition to different kinds of wood, agriculture residues and bioenergy crops, suitability of NIR spectroscopy for compositional evaluation has also been explored using flax and prairie grass [10]. However, traditional NIR spectroscopy applied for biomass energy conversion processes captures small point sources of spectrum information, but does not provide spatial dimension information. In contrast, hyperspectral imaging can simultaneously obtain spatial and spectral information from the same object. Spectral information associated with each pixel in a hyperspectral image can be used to characterize the analytical composition at the pixel level, thus allowing visualization of their spatial distribution in the sample. However, the large size of such hyperspectral data sets often complicates the process of predicting the value of a dependent variable [11, 12]. One way to overcome this problem is to implement the hyperspectral data in conjunction with variable selection to reduce the complexity of data for producing better prediction and a simpler process [13]. Thus, development of a multispectral imaging system which would be faster and cost-effective based on the variable selection performed is necessary for online automated quality control. As a result, hyperspectral imaging techniques have the potential for more precise and comprehensive information extraction for the bioenergy industry than is feasible with other techniques. To the best of our knowledge, no studies have presented a precise model for assessing the lignocellulose constituents in selection of biofuel pellets for HTC processes based on NIR hyperspectral imaging.

Considering the abovementioned background, the objects of our work follow: (1) to explore the feasibility of NIR hyperspectral imaging for determining the lignocellulose components of biofuel pellets; (2) to determine the important wavelengths for predicting cellulose, hemicellulose and lignin contents using the successive projections algorithm (SPA); (3) to develop optimal prediction models for determination of lignocellulose components; and (4) to visualize the component concentrations in the sample by transferring these models to each pixel on the hyperspectral images for online application.

## Methods

### Materials and determination of lignocellulose components

A total of 148 biofuel pellet samples were purchased from different bioenergy enterprises across China. The pellets were mainly compressed from agriculture residues (rice straw, corn stover and rice husk), forest residues (rubber, pinus, tea and mahogany) and furniture waste.

Of each sample, 10 g was ground to powder for laboratory chemical analysis of lignocellulosic substrates. The biofuels pellets were dried, and then crushed into power in a grinder (ZSJD, Linda mechanism Co., Ltd., Zhejiang, China) with a size range of 0.5–1.2 mm. The lignocellulose components of biofuel pellets (percentages of cellulose, hemicellulose and lignin) were determined using the methods of Xue et al. [9], which were originally obtained from Standard Biomass Analytical procedures and American Society for Testing and Materials procedures # NREL/TP-510-42618 [14] and ASTM E1721-01 [15].

### Hyperspectral imaging acquisition and calibration

**Fig. 1:**
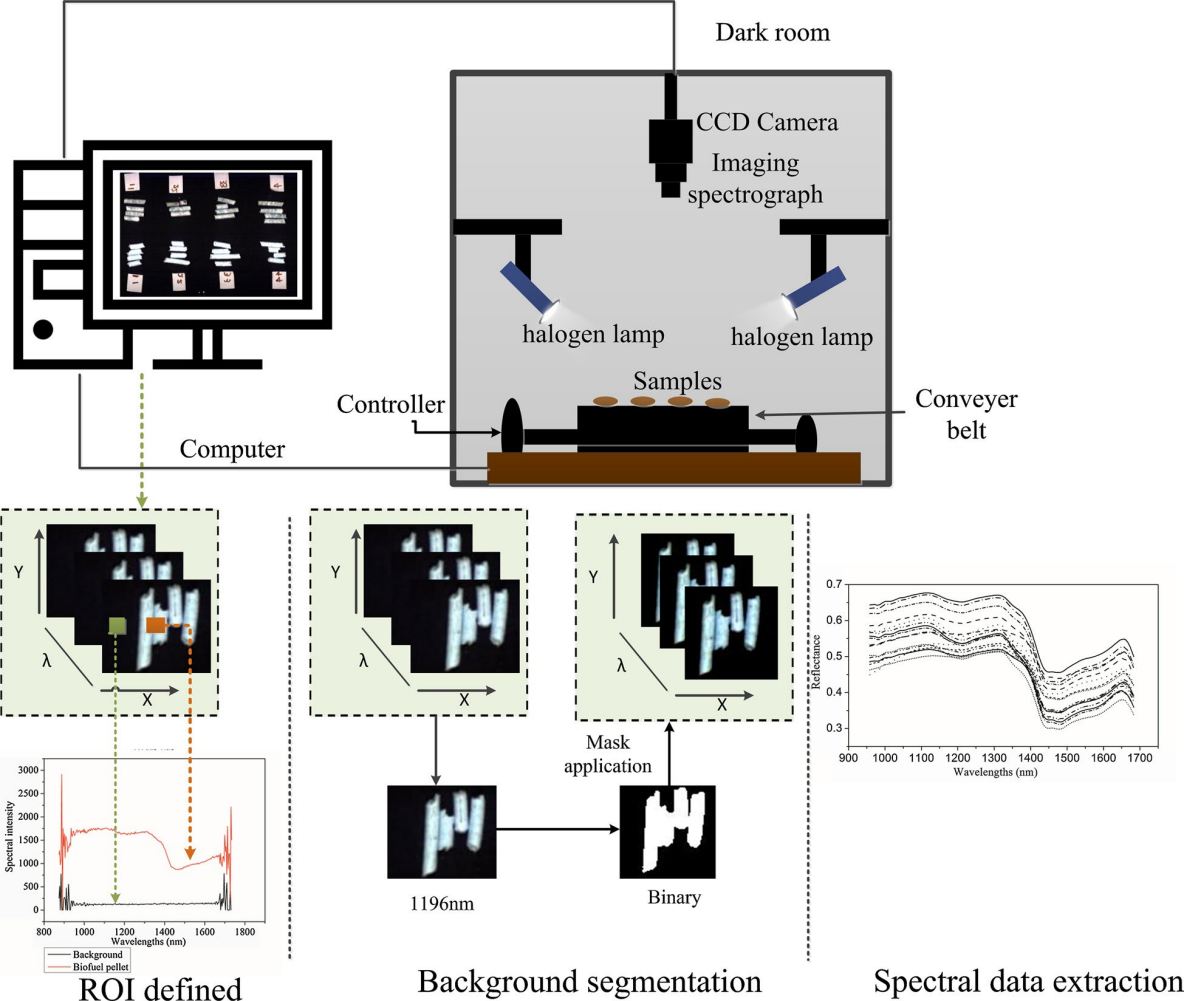
Configuration of the hyperspectral imaging system and flowchart of hyperspectral image segmentation

For each biofuel pellet sample, about 10 g was selected for hyperspectral image acquisition. The apparatus of NIR hyperspectral imaging for acquiring the images was as described by Feng et al. [16]. Figure 1 illustrates the typical push-broom system. The instruments included an ImSpector N17E imaging spectrograph (Specim, Oulu, Finland) using the wavelengths 900–1700 nm, two 150-W halogen lamps (Fiber-Lite DC9500 Illuminator; Dolan Jenner Industries Inc., USA), a CCD camera (Xeva 992; Xenices Infrared Solutions, Leuven, Belgium) and a C-mount imaging lens (OLES22; Specim). Three instrument parameters had to be reset before obtaining a clear and non-deformed image. In the present study, biofuel pellets were placed on the conveyer belt and moved at the speed of 3.1 mm/s. The exposure time of the camera was 0.03 s. The vertical distance between lens and samples was 31.2 cm. Each raw hyperspectral image of a biofuel pellet contained 256 contiguous wavelength bands and 256 congruent sub-images.

The raw hyperspectral images (*I*_raw_) were calculated using a white (*I*_white_) and a dark reference image (*I*_dark_) to correct the reflectance spectrum from the illumination and device response. The calibrated image (*I*_cal_) was calculated using Eq. (1):

1 $$I_{\text{cal}} = \frac{{I_{\text{raw}} - I_{\text{dark}} }}{{I_{\text{white}} - I_{\text{dark}} }},$$

where *I*_dark_ was acquired by turning light sources off and covering the camera with reflectance close to 0%, and *I*_white_ was obtained using white Teflon with ~ 99% reflectance under the same condition.

### Spectra extraction and data pre-processing

Each biofuel pellet sample was isolated from the background by employing the image segmentation approach with ENVI software (Version 4.6, ITT Visual Information Solutions, Boulder, CO, USA). The procedures of extracting spectral information of samples are shown in Fig. 1. Initially, a threshold for excluding any interfering information from the background was set according to the spectral differences between background and sample (spectral intensity of sample > 800 and spectral intensity of background < 800 at 1196 nm). The maximum spectral intensity value is 4095 in hyperspectral image. Segmentation was produced for detecting the biofuel pellet based on simple thresholding at a value of 0.19 (0.19 = 800/4095). Spectral information was extracted from the isolated sample portion defined as the region of interest (ROI). Each sample spectrum was an average from all the pixels spectra in each relative ROI.

Pre-processing of NIR spectral data is an important step before implementing the chemometric models for quantitative analysis. Four pre-treatment algorithms were employed to decrease any inappropriate information: mean centering (MC), standard normal variate (SNV), second derivatives (2nd derivatives) and multiplicative scatter correction (MSC) [17, 18]. All pre-processing programs were carried out with the aid of Unscrambler X Version 10.1 software (CAMO AS, Oslo, Norway).

### Development and analysis of NIR models

Sample set partitioning based on joint *x*–*y* distances (SPXY) algorithms [19] were implemented to divide 148 samples into calibration and prediction sets at a ratio of 3:1 based on their differences in both spectra and chemical composition. NIR spectra contain robust information related to chemical composition and molecular structure that is not directly available from their spectral reflectance curve results [20]; thus a chemometric approach including wavelength selection approaches and multivariate models were also implemented for spectra analysis. Hyperspectral images contain a high-dimensional dataset with a number of highly correlated variables. Therefore, a feature selection method named the SPA was applied to identify important variables for constructing cost-effective and simple models. SPA, a forward feature selection tool, adopts a simple projection operation to acquire subsets of variables with minimal collinearity [21, 22]. The SPA method was developed using the SPA toolbox of MATLAB R2010b (The Math Works, Natick, MA, USA). Here, the parameters of minimum and maximum numbers of variables selected in the SPA procedure were 5 and 50, respectively. Statistical models can build a relationship between spectral fingerprint and chemical components. To evaluate the sensitivity of variables selected by SPA, statistical models constructed using whole wavelengths and features were analyzed and compared. In the present study, partial least-squares regression (PLSR) [23] and principal component multiple linear regression (PC-MLR) [24] were implemented to build the prediction models.

The PLS algorithm projects the raw variable into a new space with new orthogonal variable called latent variables (LVs), but the first few LVs contain the most important information. The optimal LV number was determined by implementing leave-one-out cross-validation to the calibration set. PLSR was used to develop the bilinear model with the aid of Unscrambler X Version 10.1.

Multiple linear regression is suitable for the situation where the number of variable is less than the sample size. Due to the presence of multicollinearity, the standard errors of the parameter could be high [25]. The specific goals of PC-MLR are to reduce a large number of predictor variables to smaller number of principal components (PCs). In the present study, PC-MLR is used for forecasting the dependence of a response variable with PCs as inputs to reduce the model complexity and eliminate data collinearity. The optimal number of PCs was determined by the calibration model performance. The PC-MLR model was constructed using MATLAB R2010b.

Four criteria were used to evaluate the accuracy of the regression models developed: the coefficients of determination from the calibration ($R_{\text{c}}^{2}$) and prediction sets ($R_{\text{p}}^{2}$), and root mean square errors of the calibration (RMSE_C_) and the prediction sets (RMSE_P_). Ideally, a prediction model should have high $R_{\text{c}}^{2}$ and $R_{\text{p}}^{2}$, and low RMSE_C_ and RMSE_P_.

### Chemical imaging build

In addition to rapid and accurate determination of composition in the samples, another benefit of hyperspectral imaging is to display the spatial distribution of constituents and the concentration gradients of different constituents in the samples with the aid image processing [26]. One advantage of hyperspectral imaging is that it allows for quantizing different chemical composition on the “distribution map” of a sample based on their spectral fingerprint associated with each pixel in the hyperspectral image [11]. Regions of similar spectral information in the hyperspectral image should have similar biochemical constituents. Therefore, it is possible to evaluate biomass lignocellulose components from sample to sample or even with the sample using such a distribution map. The optimal calibration model for each biochemical parameter was selected after comparing the performance of statistical models previously constructed. Lignocellulose components of each pixel were predicted by transferring their optimal models to the spectrum of each pixel on the examined hyperspectral image. The visualization of chemical composition was developed in MATLAB R2010b.

## Results and discussion

### Spectral data pre-processing and analysis

The statistical results of hemicellulose, cellulose and lignin contents in both sets are shown in Table 1. Different feedstock resources had different concentrations of hemicellulose, cellulose and lignin. Compared to prediction sets, the calibration sets had a lower mean value of lignocellulose components, and higher or similar standard deviation. The ranges of hemicellulose, cellulose and lignin in the calibration set were 18.94–64.56%, 11.12–30.98% and 13.87–29.12%, respectively; and corresponding components in the prediction set were 21.44–61.00%, 11.98–30.56% and 14.32–27.24%.

**Table 1:** Statistical description of cellulose, hemicellulose and lignin concentrations for calibration and prediction sets

Indices	Cellulose (%)		Hemicellulose (%)		Lignin (%)	
	Calibration	Prediction	Calibration	Prediction	Calibration	Prediction
Number	111	37	111	37	111	37
Maximum	64.56	61	30.98	30.56	29.12	27.24
Minimum	18.94	21.44	11.12	11.98	13.87	14.32
Mean	49.14	49.31	18.42	18.5	20.25	20.28
Standard deviation	7.72	8.55	3.76	3.76	3.57	3.57

### Optimization of pre-processing methods

**Fig. 2:**
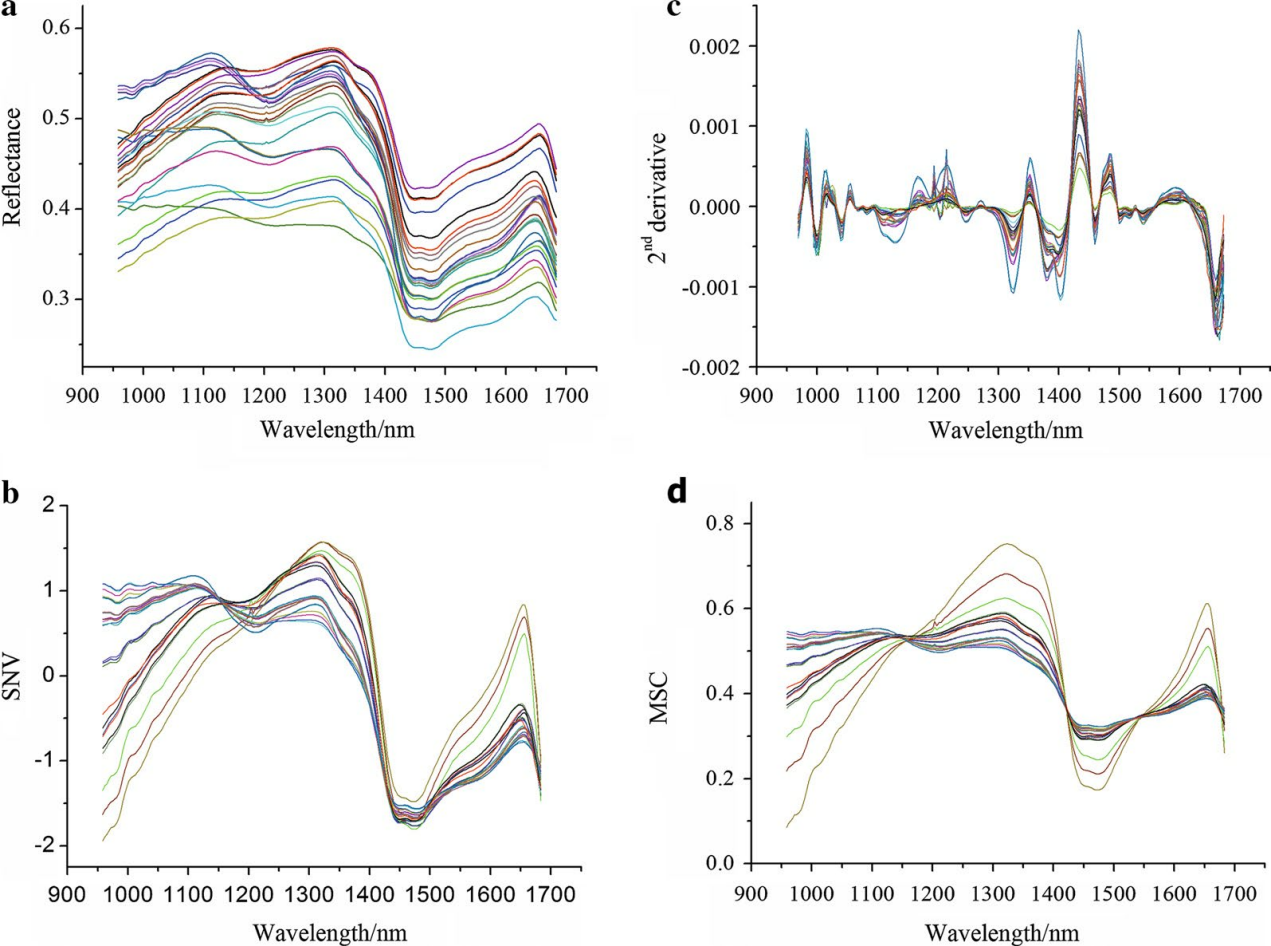
Reflectance obtained with no pre-treatment and three pre-processed spectra within wavelengths 958–1683 nm. **a** No pre-treatment; **b** standard normal variate (SNV); **c** second derivative (2nd derivative); and **d** multiplicative scatter correction (MSC)

Prior to further spectrum analysis, the front–back end range of NIR spectra (874.41–954.88 and 1686.07–1733.91 nm) was removed due to having artificial noise from the instrument and illumination (Additional file 1: Figure S1). A composite absorbance spectrum was computed for each by applying a mean centering spectral pretreatment on the individual biofuel pellet spectrum to bring the spectral to a common axis, and the averaging the 148 spectra. However, MC pretreatment was not helpful for improving the predictive capability of the models (Additional file 2: Table S1). NIR spectral pre-processing methods including 2nd derivatives, SNV and MSC were implemented in order to decrease the effects from physical and chemical interferences and enhance the predictive ability of mathematical models (Fig. 2). The impact of pre-processing strategies on predictive accuracy was compared by constructing multivariate calibration models using PLSR and PC-MLR. In this step, PLSR and PC-MLR predictive models for determination of chemical components were established on the whole wavelengths. The newly proposed combined mathematical models were assessed and compared: raw–PC-MLR, SNV–PC-MLR, 2nd derivative–PC-MLR, MSC–PC-MLR, raw–PLSR, SNV–PLSR, 2nd derivative–PLSR and MSC–PLSR (Table 2).

**Table 2:** Prediction results of the pre-processing models constructed by partial least-squares regression (PLSR) and principal component multiple linear regression (PC-MLR) for lignocellulose components of biomass pellets

Indices	Model type	Pre-processing	Par^a^	Calibration set		Prediction set	
				$R_{\text{p}}^{2}$	RMSE_C_ (%)	$R_{\text{p}}^{2}$	RMSE_P_ (%)
Cellulose	PC-MLR	Raw	29	0.93	2.21	0.91	2.49
		SNV	21	0.86	3.19	0.87	2.95
		2nd	45	0.93	2.26	0.79	3.82
		MSC	21	0.84	3.39	0.85	3.27
	PLSR	Raw	10	0.91	2.64	0.91	2.51
		SNV	11	0.84	3.36	0.83	3.39
		2nd	2	0.61	5.23	0.61	5.23
		MSC	10	0.83	3.56	0.81	3.63
Hemicellulose	PC-MLR	Raw	21	0.83	1.54	0.79	1.68
		SNV	16	0.81	1.61	0.83	1.53
		2nd	45	0.87	1.36	0.78	1.74
		MSC	18	0.80	1.66	0.78	1.73
	PLSR	Raw	12	0.82	1.54	0.80	1.86
		SNV	10	0.81	1.60	0.82	1.58
		2nd	9	0.83	1.56	0.76	1.83
		MSC	7	0.72	1.95	0.59	2.67
Lignin	PC-MLR	Raw	38	0.88	1.19	0.75	1.75
		SNV	21	0.71	1.90	0.60	2.23
		2nd	32	0.85	1.30	0.74	1.78
		MSC	13	0.61	2.23	0.52	2.44
	PLSR	Raw	13	0.86	1.31	0.74	1.79
		SNV	8	0.61	2.20	0.46	2.58
		2nd	16	0.82	1.47	0.71	1.87
		MSC	6	0.58	2.28	0.45	2.61

^a^ Model parameters indicate the optimal number of latent variables for establishing the PLSR calibration model and optimal number of principal components for PC-MLR; $R_{\text{c}}^{2}$ and $R_{\text{p}}^{2}$, coefficients of determination for calibration and prediction sets, respectively; RMSE_C_ and RMSE_P_, root mean square errors of calibration and prediction sets, respectively; SNV, standard normal variate; 2nd, second derivative; MSC, multiplicative scatter correction

For cellulose, pre-treatment of NIR spectra data did not improve the predictive capability of models for both PLSR and PC-MLR. The best cellulose prediction model was developed by PC-MLR using no spectral pre-processing before regression analysis (i.e., raw–PC-MLR), with $R_{\text{c}}^{2}$, RMSE_C_, $R_{\text{p}}^{2}$ and RMSE_P_ of 0.93, 2.21, 0.91 and 2.49%, respectively. The strongest hemicellulose prediction model was SNV–PC-MLR with corresponding values of 0.81, 1.61, 0.83 and 1.53%; and the SNV–PLSR model for hemicellulose slightly decreased its predictive capability compared with SVN–PC-MLR, with 0.81, 1.60, 0.82 and 1.58%. Similarly, the pre-processing did not improve the prediction performance of PLSR and PC-MLR model for lignin. The preferred mathematical model for the prediction of lignin was raw–PC-MLR with $R_{\text{c}}^{2}$ = 0.88, RMSE_C_ = 1.19%, $R_{\text{p}}^{2}$ = 0.75 and RMSE_P_ = 1.75% (Table 2).

Cellulose is the most abundant and critical polysaccharide for plant biomass. Therefore, the cellulose prediction model had better performance than those for hemicellulose and lignin, consistent with previous results [8, 9, 27]. In the present study, the capability of predicting biomass pellet lignocellulose components for the PC-MLR model was better than for the PLSR when the whole NIR spectral data were used. In addition, spectra data pre-treatment was only helpful for prediction of hemicellulose. Thus, NIR spectral data pretreated by SNV for hemicellulose determination, and spectral data without pre-treatment for cellulose and lignin determination were used for further analyses. Successful application of NIR spectra combined with chemometric methods for the determination of lignocellulose components of biomass energy was also demonstrated in previous studies [8, 9, 20]. Jin et al. [8] collected the NIR spectral of biomass crop *Miscanthus* with the spectral rang of 400–2500 nm and established PLS determination models for hemicellulose, cellulose and lignin, respectively, and the results were $R_{\text{p}}^{2}$ of 0.93 and RMSE_P_ of 0.71% for hemicellulose; $R_{\text{p}}^{2}$ of 0.94 and RMSE_P_ of 0.68% for cellulose; and $R_{\text{p}}^{2}$ of 0.86 and RMSE_P_ of 0.56% for lignin. Wang et al. [28] reported PLSR models with *R*^2^ of 0.53 and RMSE of 0.73 in the calibration sample set for hemicellulose; *R*^2^ of 0.56 and RMSE of 0.76 for cellulose; *R*^2^ of 0.56 and RMSE of 1.23 for lignin at the wavelength range of 830–25000 nm for soybean straw. Our NIR model performance established on shorter wavelength range of 958–1683 nm for predicting lignocellulose components was comparable to or better than previous works that predicted such content in biomass crop.

### Selection of optimal wavelengths

**Fig. 3:**
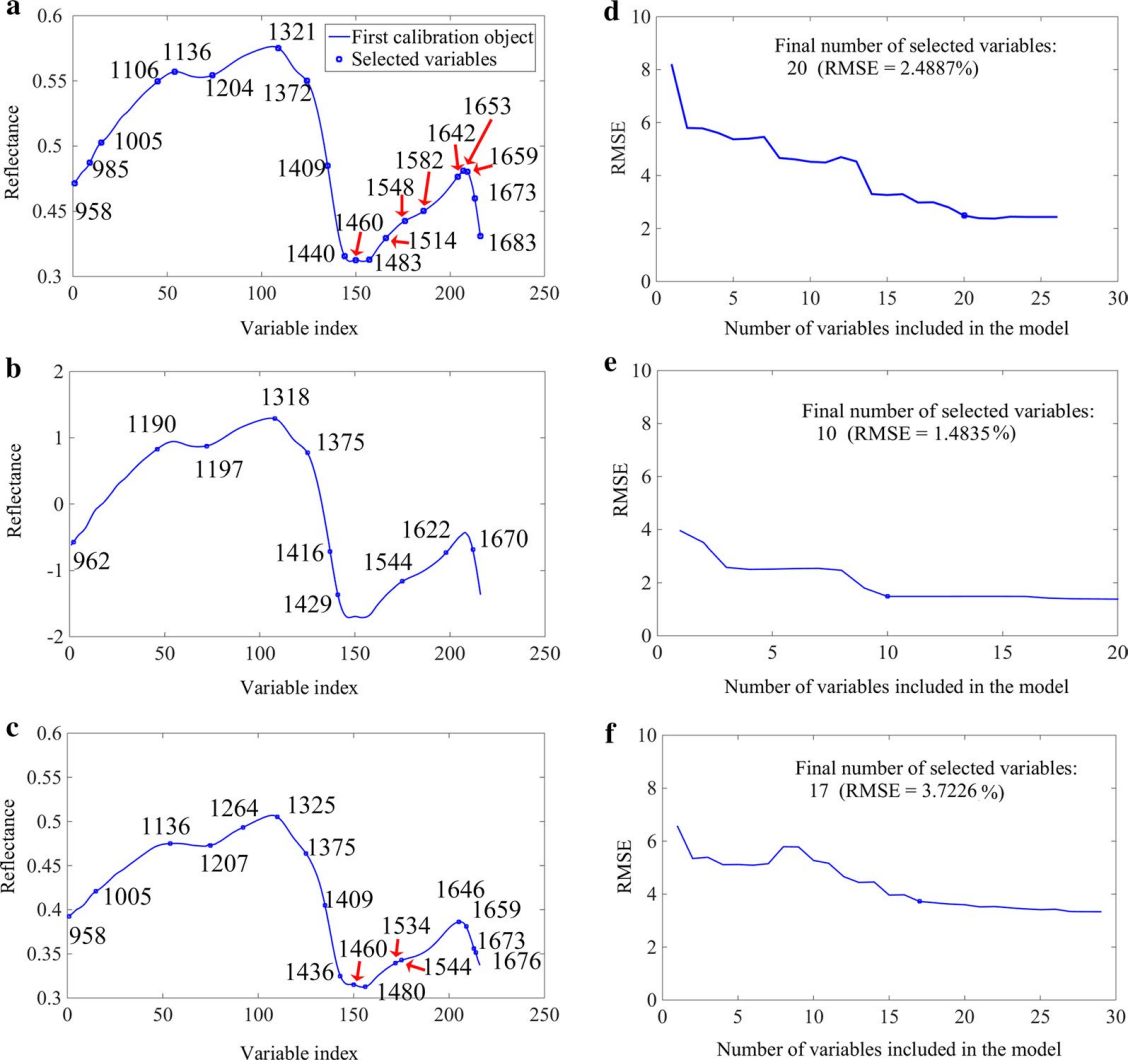
Selection of optimal wavelengths by successive projections algorithm. Distributions of important variables (marked with ‘filled circle’) for cellulose (**a**), hemicellulose (**b**) and lignin (**c**); final number of selected variables for cellulose (**d**), hemicellulose (**e**) and lignin (**f**) determined on the basis of the root mean square error (RMSE) of validation set of multiple linear regression models

A statistical model established using a number of variables that are highly correlated would increase the computational complexity of prediction [29]. Thus, the selection step for sensitive wavelengths in multivariate analysis is necessary to determine the most important spectral information and simplify the process. SPA seemed to be the better method to save the multicollinearity problems compared to other method (Additional file 3: Figure S2). Therefore, reduced variables selected by SPA were set as the input variable to build multivariate models by PLSR and PC-MLR for predicting lignocellulose component in this study. SPA was proposed to identify the characteristic wavelengths for the determination of cellulose, hemicellulose and lignin; and the number of wavelengths selected by SPA decreased by 9.26, 4.63 and 7.87%, respectively (Fig. 3).

In general, absorptions at the optimal wavelengths are closely related to molecular structures of the chemical components. Some wavelengths (1321, 1372, 1548 and 1673 nm) were shared by cellulose, hemicellulose and lignin (Fig. 3), indicating similar molecular structures among these chemical components. The absorption band at ~ 1320 nm may be assigned to C–H combination related to CH_2_ [30]. The other wavelengths bands were primarily attributed to the first overtone of C–H stretching and/or deformation (1372 nm), first overtone of O–H stretching (1548 nm) and first overtone of aromatic C–H stretching (1673 nm) [31]. Individual absorption bands that were sensitive for cellulose mainly corresponded to the second overtone of O–H vibration (985 nm); second overtones of C–H stretching (1204 nm); first overtone of C–H stretching and/or deformation of CH, CH_2_ and CH_3_ groups (1440 and 1683 nm); first overtone of O–H stretching from cellulose (1483, 1514 and 1582 nm); and first overtone of C=C from vinyl group (1653 and 1659 nm) [31–33]. For hemicellulose, the characteristic wavelengths of 1109, 1197, 1416, 1429 and 1622 nm are connected with the combination of O–H stretching (first overtone) [31]. The special signal bands that are sensitive for lignin include 1264, 1534 and 1646 nm. The absorption at 1264 nm is connected to the third overtone of C–H harmonic stretching [34]. The signal at ~ 1534 nm is assigned to the first overtone of O–H stretching [31]. The absorption band near 1646 nm may be due to the aliphatic C–H vibrations and first and second overtones of lignin aromatic rings [35].

### Establishment of multivariate models based on optimal wavelengths

**Fig. 4:**
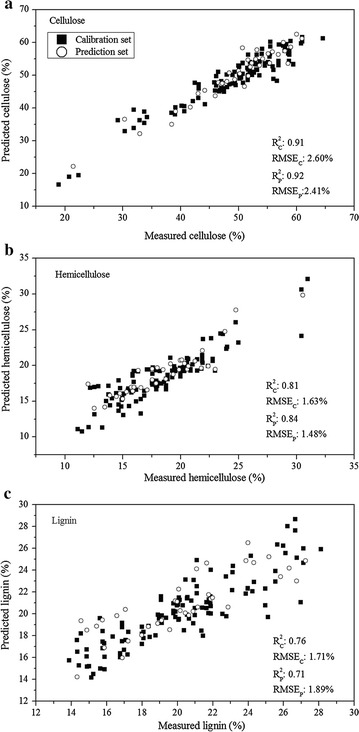
Performance of best prediction models for determination of biofuel pellet lignocellulose components based on characteristic wavelengths. **a** SPA–PC-MLR model for cellulose; **b** SNV–SPA–PC-MLR model for hemicellulose and **c** SPA–PC-MLR model for lignin

After reducing the amount of spectral data into several variables by using the wavelength selection method, NIR band assignments provide some insight into the relationship between spectral features and chemical components. The whole spectral data set was decreased to a matrix of dimensions *m* × *n*, where *m* represents the number of samples (*m* = 148) and the number of wavelengths *n* was 20 for determination of cellulose, 10 for hemicellulose and 17 for lignin. To inspect the suitability of optimal variables selected by SPA, the sensitive wavelengths were set as the input variables to build multivariate models by PLSR and PC-MLR for predicting lignocellulose components. Table 3 shows the performance of prediction models established on the characteristic wavelengths. The newly introduced models were compared and evaluated: SPA–PLSR, SPA–PC-MLR, SNV–SPA–PLSR and SNV–SPA–MLR. The PC-MLR models established on characteristic wavelengths for the prediction of cellulose, hemicellulose and lignin were again better than the PLSR models. The $R_{\text{c}}^{2}$ for cellulose, hemicellulose and lignin were 0.91, 0.81 and 0.76 with RMSE_C_ of 2.60, 1.63 and 1.71%, respectively; whereas the corresponding values for $R_{\text{p}}^{2}$ were 0.92, 0.84 and 0.71 and for RMSE_P_ were 2.41, 1.48 and 1.89%. The best-performing prediction models for determining the compositional contents according to variable selection methods were the SPA–PC-MLR for cellulose and lignin and the SPA–SNV–PC-MLR for hemicellulose (Fig. 4). PC-MLR models for the contents of cellulose and hemicellulose calculated on optimal wavelengths had similar accuracy to corresponding models based on the whole spectrum (Tables 2 and 3). This indicates that the characteristic wavelengths were the primary contributors for construction of cellulose and hemicellulose determination models. However, the SPA–PC-MLR model for prediction of lignin had worse performance, with relatively lower $R_{\text{c}}^{2}$ and $R_{\text{p}}^{2}$, compared with the model using the full spectral data (Fig. 4c).

**Table 3:** Result of PLSR and PC-MLR models for cellulose, hemicellulose and lignin based on optimal wavelengths

Indices	Model type	Par^a^	Calibration set		Prediction set	
			$R_{\text{c}}^{2}$	RMSE_C_ (%)	$R_{\text{p}}^{2}$	RMSE_P_ (%)
Cellulose	SPA–PC-MLR	18	0.91	2.60	0.92	2.41
	SPA–PLSR	11	0.90	2.77	0.91	2.52
Hemicellulose	SNV–SPA–PC-MLR	10	0.81	1.63	0.84	1.48
	SNV–SPA–PLSR	8	0.79	1.69	0.80	1.63
Lignose	SPA–PC-MLR	16	0.76	1.71	0.71	1.89
	SPA–PLSR	12	0.75	1.76	0.65	2.06

^a^Similar model parameters and abbreviations as in Table 2. *SPA* successive projections algorithm

Xun et al. [9] established PLSR models for prediction of corn stover lignocellulose components with spectral range of 522–1567 nm: the prediction results were $R_{\text{p}}^{2}$ of 0.77 and RMSE_P_ of 15.28 g/kg for cellulose; and correspondingly 0.62 and 9.47 g/kg for hemicellulose, and 0.61 and 11.73 g/kg for lignin. Huang et al. [27] collected NIR spectral data of 172 rice straw samples and built PLSR models for determining the lignocellulose components at the wavelength range of 400–2500 nm, and obtained more accurate results (validation results of *R*^2^ were 0.82, 0.71 and 0.78 for cellulose, hemicellulose and lignin, respectively). Compared with results of Xue et al. [9] and Huang et al. [27], our prediction accuracy for cellulose and hemicellulose was greatly increased. Moreover, less than 10% of the independent variables were used in this study due to application of the optimal wavelength selection method. This dimension reduction significantly raises computer processor speed and simplifies the prediction models. By extension, appropriate reductions in numbers of independent variables offers a promising alternative for developing real-time multispectral instruments for online industry application. Thus, SPA–PC-MLR for cellulose and lignin and the SNV–PC-MLR for hemicellulose models were used to construct distribution maps in the next step.

### Construction of distribution maps for lignocellulose components

**Fig. 5:**
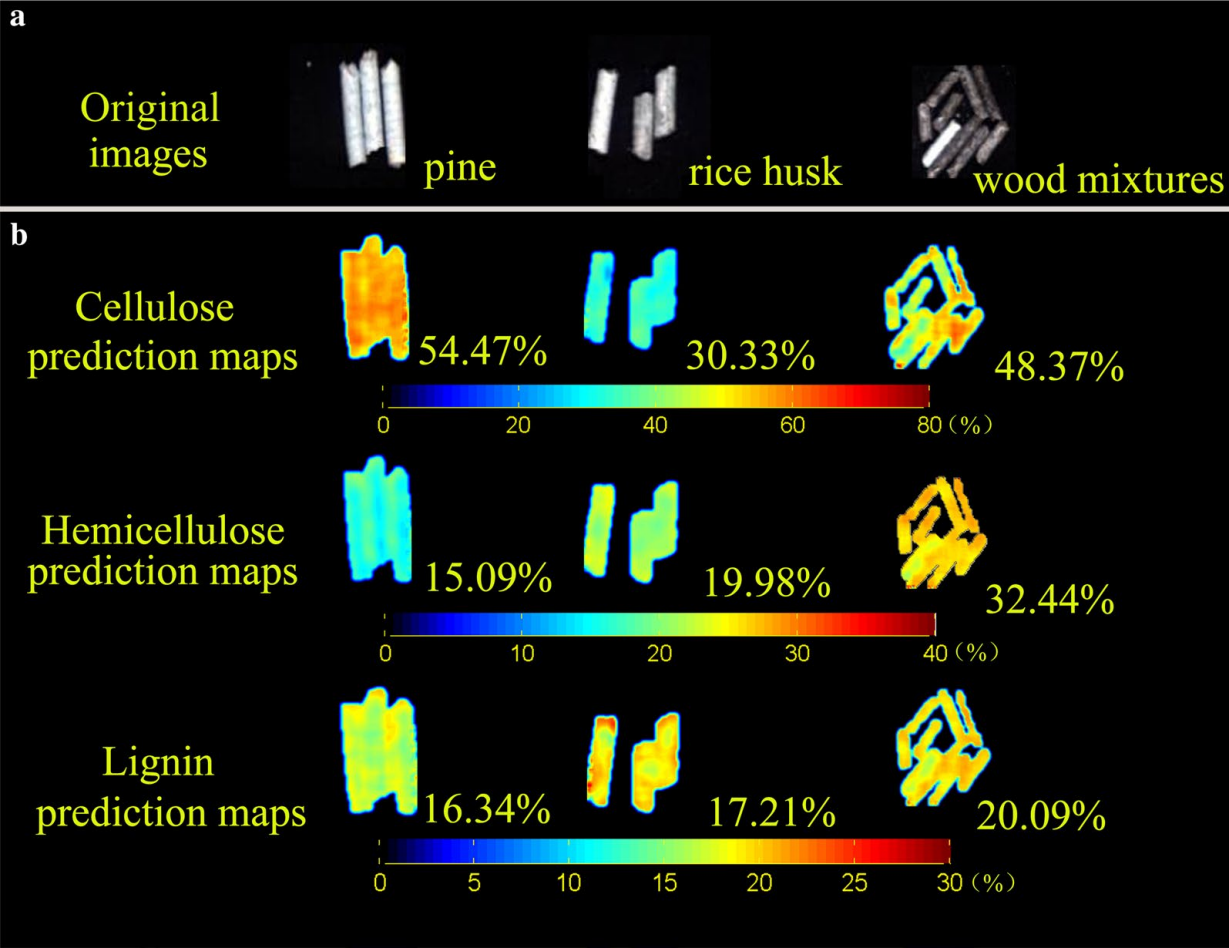
Distribution maps of cellulose, hemicellulose and lignin contents in different biofuel pellets. **a** Original biofuel pellet NIR images; **b** prediction map of different lignocellulose components. The numbers accompanying each sample represent the respective lignocellulose component content. The three color-scale bars were generated with different cellulose, hemicellulose and lignin contents from small to large, shown in different colors from blue to red

At the final step of hyperspectral image analysis, the optimal simplified models were applied to produce cellulose, hemicellulose and lignin distribution maps among and within the biofuel pellets at the pixel level. All pixel features were predicted by implementing the best-performing model at the examined hyperspectral image. A median filter technique was used for removing salt-and-pepper noise during the imaging processing program [36]. Figure 5 shows the lignocellulose component distribution map for different kinds of biofuel pellets. Different colors shown on the distribution map represents different parameters values, which correspond to different spectral features of pixels. Although it is impossible to determine the contents of lignocellulose components in the different biofuel pellet samples in the original NIR image (Fig. 5a), the spatial variation of these parameters among the variety of pellets can be visualized in the generated distribution maps (Fig. 5b). The major components of biomass pellets vary according to the biomass feedstock and production process, which in turn significantly influence the final conversion processing strategies. Wood biofuel pellets had higher cellulose (48.37% for wood mixtures and 54.47% for pine) than herbaceous feedstocks such as rice husk (30.33%); and rice husk biomass had much higher hemicellulose concentration (19.98%) than pine wood (15.09%). The biofuel pellet size, which affects pellet durability, could also be detected simultaneously by image processing means. Hyperspectral imaging can obtain spectral and spatial features from an object, which are both important for employing automatic approaches to biomass quality assurance and control. The number of analytical compositions can be rapidly and simultaneously visualized from the spatial distribution map. This might be important in determining suitable candidates for further HTC treatment and improving management of pellet production. However, the lignin prediction model could be enhanced by including more samples with a large range of related values. The biomass pellet industry and market could benefit from the results presented by using hyperspectral imaging for fast and accurate determination of biomass composition in biofuel pellets to accelerate biomass utilization and improve the procedure control.

## Conclusions

Lignocellulose biomass has been proposed as an option for production of chemicals and fuels because it could reduce greenhouse gas emission by substituting for petroleum fuels. The major components of lignocellulosic biomass are cellulose, hemicellulose and lignin. The chemical composition and physical architecture of biofuel pellets influences the process control in the selection conversion technology and characteristics of final product. The capability of hyperspectral imaging in monitoring lignocellulose components of biofuel pellets in its ability to provide spectral information related to molecular structures of the chemical components. In addition, multispectral imaging systems are also suggested to be developed and applied for online application. Rapid and accurate measurement of biomass composition is important for increasing biomass utilization, and so the lignocellulose components of biofuel pellets were determined using an online hyperspectral imaging system. The results demonstrated that the hyperspectral imaging technique coupled with chemometric analysis such as SPA and PC-MLR were rapid and non-destructive, and accurately predicted the cellulose, hemicellulose and lignin contents of biomass pellets. Out of 216, only 20, 10 and 17 wavelengths were identified as important by SPA and found to be suitable for corresponding biochemical composition determination. The SPA–PC-MLR calibration model acquired good results of $R_{\text{p}}^{2}$ = 0.92 and RMSE_P_ = 2.41% for cellulose and $R_{\text{p}}^{2}$ = 0.71 and RMSE_P_ = 1.89% for lignin. The SNV–SPA–PC-MLR gave a satisfactory result of $R_{\text{p}}^{2}$ = 0.84 and RMSE_P_ = 1.48% for hemicellulose. To the authors’ knowledge, this is the first publication of the distribution of lignocellulose components of biofuel pellets at the pixel level and demonstrates how concentrations differ among samples. One benefit of the wavelength selection method is that the visualized map generated on reduced independent variables will help in developing a convenient and low-cost multispectral imaging device for online industry application. In the future, more biofuel pellets with a wide range of lignocellulosic composition should be studied to established more accurate and robust inspection model which could be applied in the biomass production industry. On the other hand, other variable selection methods should be considered to selection best wavelengths with higher accuracy and fewer numbers to inspect the content and distribution of lignocellulose components and other components of biofuel pellets. Furthermore, more attention should be paid on the physical specifics (shape, density and others) of different variety on the impact of hyperspectral imaging analysis in future investigation.

## Additional files

**Additional file 1: Figure S1.** Profiles of reference spectra for biofuel pellets extracted from raw near-infrared hyperspectral images.

**Additional file 2: Table S1.** Result of PLSR models for cellulose, hemicellulose and lignin based on raw spectra and mean centering pretreatment.

**Additional file 3: Figure S2.** Selected important wavelengths by competitive adaptive reweighted sampling (CARS). 39 wavelengths for cellulose; 29 wavelengths for hemicellulose and 26 wavelengths for lignin. The number of Monte Carlo sampling runs was set to 50, and tenfold cross-validation was used to evaluate the effectiveness of each subset of variables with a number of highly correlated variables.

## Abbreviations

HTC: hydrothermal carbonization
NIR: near-infrared
*I*_raw_: the raw hyperspectral images
*I*_white_: the white reference image
*I*_dark_: the black reference image
*I*_cal_: the calibrated image
ROI: region of interest
SNV: standard normal variate
2nd derivatives: second derivatives
MSC: multiplicative scatter correction
SPA: successive projections algorithm
PLSR: partial least-squares regression
PC-MLR: principal component multiple linear regression
$R_{\text{c}}^{2}$: coefficients of determination from the calibration sets
$R_{\text{p}}^{2}$: coefficients of determination from the prediction sets
RMSE_C_: root mean square errors of the calibration sets
RMSE_P_: root mean square errors of the prediction sets
SPXY: sample set partitioning based on joint *x*–*y* distances
