# Supplementary figures and images for: Nondestructive and rapid determination of lignocellulose components of biofuel pellet using online hyperspectral imaging system
Source: Biotechnol Biofuels. 2018 Apr 2;11:88. doi: 10.1186/s13068-018-1090-3 (PMC5879804; doi:10.1186/s13068-018-1090-3)

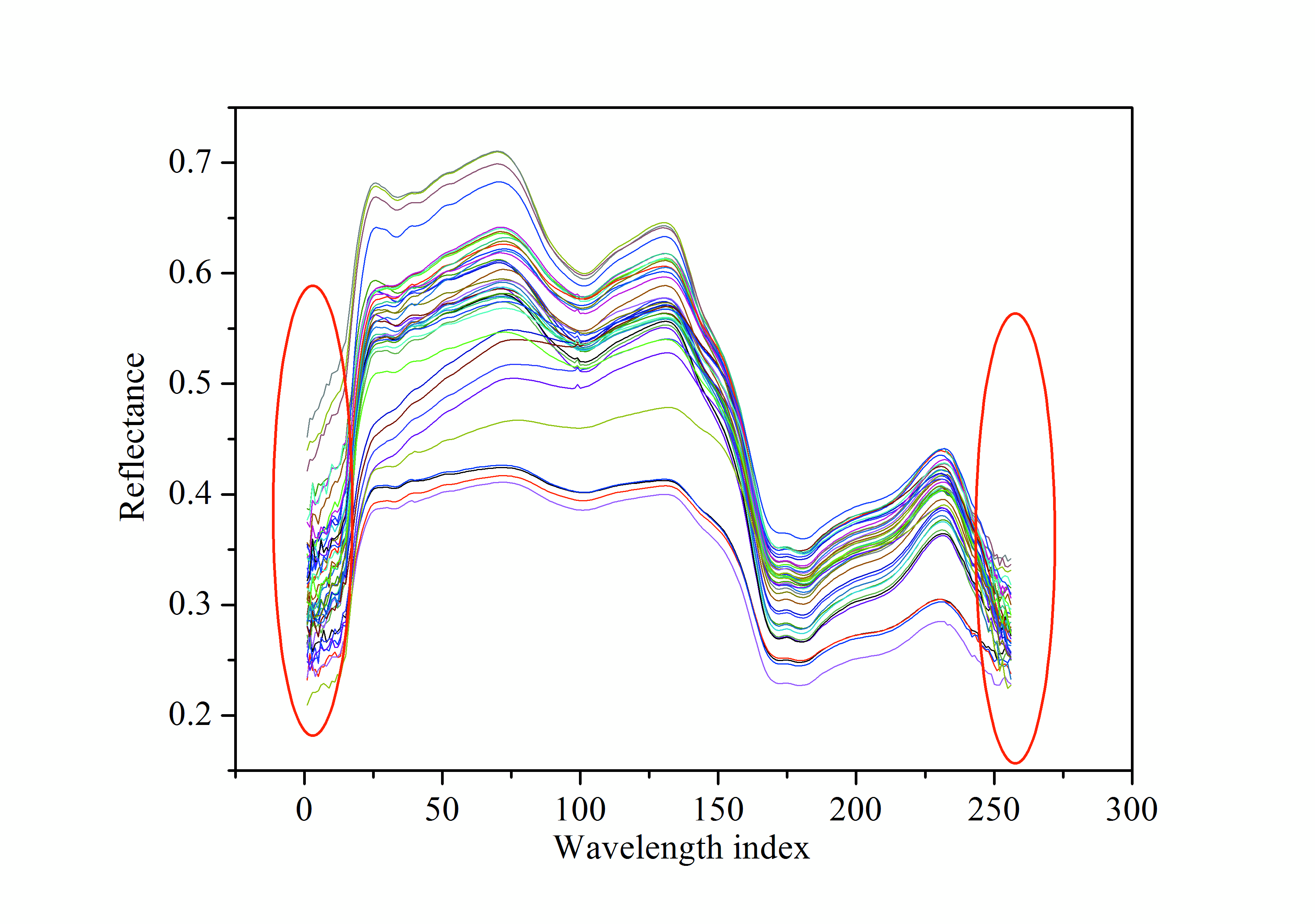

Supplement: Supplementary file 1 — Additional file 1: Figure S1. Profiles of reference spectra for biofuel pellets extracted from raw near-infrared hyperspectral images. [file 13068_2018_1090_MOESM1_ESM.tif]

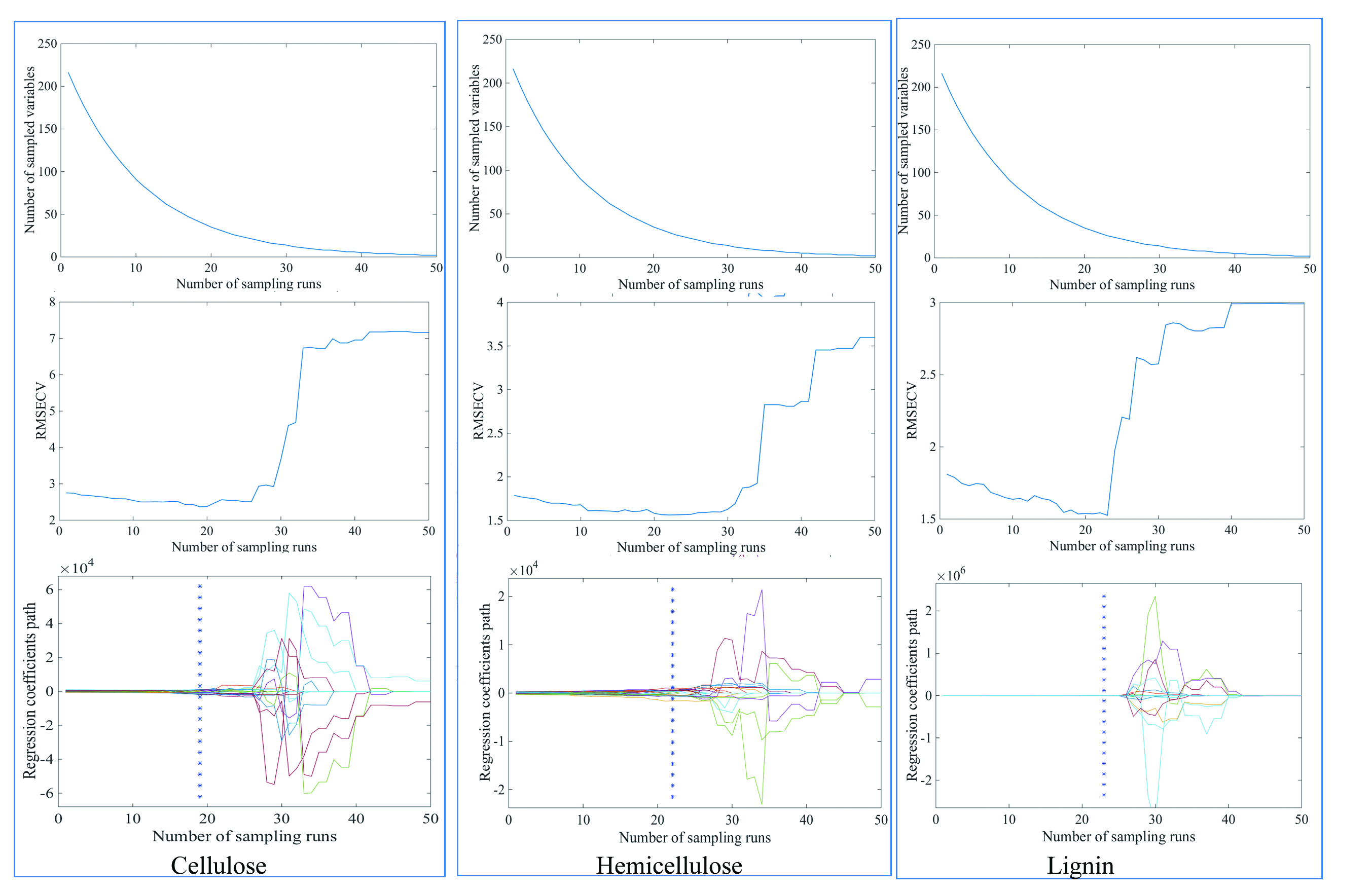

Supplement: Supplementary file 3 — Additional file 3: Figure S2. Selected important wavelengths by competitive adaptive reweighted sampling (CARS). 39 wavelengths for cellulose; 29 wavelengths for hemicellulose and 26 wavelengths for lignin. The number of Monte Carlo sampling runs was set to 50, and tenfold cross-validation was used to evaluate the effectiveness of each subset of variables with a number of highly correlated variables. [file 13068_2018_1090_MOESM3_ESM.tif]
