# Supplementary material for: Nondestructive and rapid determination of lignocellulose components of biofuel pellet using online hyperspectral imaging system
Source: Biotechnol Biofuels. 2018 Apr 2;11:88. doi: 10.1186/s13068-018-1090-3 (PMC5879804; doi:10.1186/s13068-018-1090-3)
Supplement: Supplementary file 2 — Additional file 2: Table S1. Result of PLSR models for cellulose, hemicellulose and lignin based on raw spectra and mean centering pretreatment. [file 13068_2018_1090_MOESM2_ESM.docx]

Table S1 Result of PLSR models for cellulose, hemicellulose and lignin based on raw spectra and mean centering pretreatment

| Indices | Model type | Par | Calibration set | | Prediction set | |
| --- | --- | --- | --- | --- | --- | --- |
|  |  |  | R2 c | RMSEC (%) | R2 p | RMSEP (%) |
|  | Raw–PLSR | 10 | 0.91 | 2.63 | 0.91 | 2.51 |
| Cellulose | Mean centering–PLSR | 10 | 0.91 | 2.64 | 0.91 | 2.51 |
|  |  |  |  |  |  |  |
| Hemicellulose | Raw–PLSR | 12 | 0.82 | 1.54 | 0.80 | 1.86 |
|  | Mean centering–PLSR | 10 | 0.81 | 1.61 | 0.74 | 1.89 |
| lignin | Raw–PLSR | 13 | 0.86 | 1.31 | 0.74 | 1.79 |
|  | Mean centering–PLSR | 11 | 0.80 | 1.57 | 0.67 | 2.03 |
